# Modeling and Simulation of an Energy Integrated Distillation Column in a Bond Graph Approach

**DOI:** 10.3390/e24091191

**Published:** 2022-08-25

**Authors:** Juancarlos Mendez-B, Gilberto Gonzalez-Avalos, Noe Barrera Gallegos, Gerardo Ayala-Jaimes, Carlos Rubio-Maya

**Affiliations:** 1 Graduate Studies Division of the Faculty of Mechanical Engineering, University of Michoacán, Morelia 58000, Mexico; 2 Faculty of Mechanical Engineering, University of Michoacán, Morelia 58000, Mexico; 3 Faculty of Sciences of Engineering and Technology, Autonomous University of Baja California, Tijuana 21500, Mexico

**Keywords:** bond graph, distillation column, integrated energy, junction structure, nonlinear systems

## Abstract

The bond graph methodology for modelling an integrated energy distillation column is applied in this paper. The distillation column is built by five trays for a binary mixture. However, due to its modular construction in a bond graph, the number of trays can be increased. In order to link the analysis tools of systems modeled in the bond graph to the mathematical model given to a distillation column, a junction structure of the proposed bond graph is presented. Hence, this junction structure is a way to obtain the state space representation of the modeled column in bond graphs. Likewise, it is well known that distillation columns determine a class of nonlinear systems, so throughout this paper, these systems in a bond graph approach can be analyzed. In order to learn the behavior of the distillation column in the physical domain, simulation results using 20-Sim software are shown. In addition, with the simulation of two case studies consisting of two mixtures with different relative volatilities, the versatility of the column model in a bond graph is presented. In both cases, the increase in the feed flow, the mole fraction of the light component in the feed or the distillate reflux that enriches the concentration of light in the column determine an increase in the mole fraction of light in the distillate and in the bottom reflow. Further, the control design for a distillation column in the physical domain can be extended.

## 1. Introduction

The most important separation operation in process engineering is distillation. The distillation process allows for the separation of mixtures due to the differences in their volatilities, which is achieved by the application or removal of heat. However, these heating or cooling operations require the consumption of a huge amount of energy. Distillation columns have been used in a wide number of applications, for example, from alcoholic beverages to oleo chemicals of pharmaceutical plants.

There are a large number of references on modeling, analysis and processes in distillation columns that we can cite. In [1], the modeling and simulation of a distillation column via Matlab are described. In [2], the use of Aspen and Matlab in the modeling and simulation of a batch distillation column is proposed. The modeling and control of a distillation column in a petroleum process are presented in [3]. The simulation of a segmented distillation column in flexible operation considering dynamic modeling is developed in [4]. The modeling and simulation of a batch reactive distillation column with optimization effects are proposed in [5]. Steady-state modeling of reactive distillation columns is introduced in [6]. In [7], the separation of a methanol/water mixture is developed in the ASPEN PLUS software for the modeling and simulation of the distillation column.

In order to reduce their significant energy consumption, there have been several successful developments from the use of process and energy integration techniques applied to distillation columns. The aim of these energy-integrated schemes is to reduce costs as a result of less energy consumption [8]. A new distillation column configuration constructed by the Toyo Engineering Corporation with a reduction in energy consumption is presented in [9]. The optimal design of distillation columns using an exergy graphical method is proposed in [10]. A control structure of a heat-integrated distillation column using a benzene and toluene mixture for an optimal operation is proposed in [11]. The fluid separation by distillation using a modular model framework is introduced in [12]. The limitations of distillation columns in the separation of multicomponent mixtures with different structures are considered in [13].

The modeling of a distillation column using Dymola and Simulink is found in [14]. The modeling and simulation of the connection of two distillation columns of the ethanol–water mixture in the EMSO platform is developed in [15]. The use of ASPEN PLUS in the modeling and simulation of a distillation column of the methanol/water mixture is described in [16].

There are also numerous references on the control of distillation columns that can be cited. The implications of control for integrated distillation networks are explained in [17]. Multivariable control applied to distillation columns is proposed in [18]. The control of an energy-integrated distillation column and the selection of its structure is introduced by [19]. Different control configurations applied to distillation columns with respect to conceptual design are presented in [20].

In the modeling and simulation of distillation columns, various methods have been proposed, and some software has been used. Hence, in this paper, the bond graph methodology in the modeling of a distillation column is applied.

Bond graph theory offers the ability to model systems in a structural and generalized way [21,22,23]. These systems can be linear, nonlinear, time-varying with concentrated or distributed parameters. From a bond graph model, the mathematical model of the system in a transfer function or in state space can be obtained. Various important system analysis tools such as stability, controllability, observability, linearization and steady state in bond graph models can be determined directly [24]. Furthermore, the use of the bond graph in the controller design has been extended. One of the great advantages of bond graphs is the ability to model systems composed of electrical, mechanical, hydraulic, thermal and chemical sections [25].

Therefore, modeling an integrated energy distillation column in a bond graph approach is proposed in this paper. A junction structure for the determination of the mathematical model in state space from the bond graph model of the distillation column is presented.

A bond graph model of a distillation column is proposed in [26], in which the balance equations are obtained in parts, and the validation of the mathematical model requires great effort. Some advantages of the proposed paper with respect to [26] are that the construction of the full column bond graph is clearly displayed, each stage indicates the incoming and outgoing flows, and the determination and validation of the mathematical model are performed in a structured manner.

In this paper, the modeled distillation column has five plates, but due to the modular characteristic in the construction of bond graph models, columns with a high number of plates, such as in industrial cases, can be modeled in the physical domain.

The proposed junction structure allows obtaining the state space representation of the distillation column showing the nonlinear characteristic. This structure accepts the possible derivative causality of the storage elements, that is, that it can have linearly dependent elements.

Furthermore, the relationships between the different system variables can be determined in the bond graph model, and the analysis of structural properties such as observability, controllability, linearization and steady-state can be applied.

The versatility of the bond graph is expressed with the application of the proposed methodology to the simulation of two case studies of a distillation column with different relative volatility. The graphic results of the concentrations of the light component in the distillate and bottom flow through simulations using 20 Sim software are shown. In order to demonstrate the advantages of a bond graph model for this type of process, the behavior of chemical potentials along the column is illustrated.

However, other variables can be analyzed, such as the chemical power and the relationships of the variables in the different stages of the column, which can be achieved through causal paths in bond graph models.

The novelty of this paper is the description of a bond graph model of a distillation column and the direct connection with its well-established mathematical model in various published references [27,28,29]. This allows us to state the following advantages:

- The increase in the number of stages is immediate with a greater number of elements in the bond graph.
- Other auxiliary elements of a column, such as a condenser, reboiler and preheater, can be modeled in the bond graph and included in the column model in order to know the behavior of these elements in the column variables.
- The configuration of two columns for complete extractive distillation processes is possible by connecting the bond graph models of the conventional column with the extractive column.
- Structural properties such as structural observability and controllability, linearization, steady-state and singular perturbations of bond graph modeled systems can be applied.
- Control design to a column in the bond graph approach can be achieved.

Section 2 summarizes the traditional model of a distillation column describing the elements and variables that are part of the system. The essential elements in bond graph modeling are described in Section 3. A junction structure for linking bond graph models to mathematical models for this class of nonlinear systems is proposed in Section 4. The model of a distillation column with five stages in the physical domain is presented in Section 5. Two case studies in order to obtain the simulation results of the bond graph of the distillation column are analyzed in Section 6. Finally, Section 8 presents our conclusions.

## 2. Modeling of a Distillation Column

**Figure 1:**
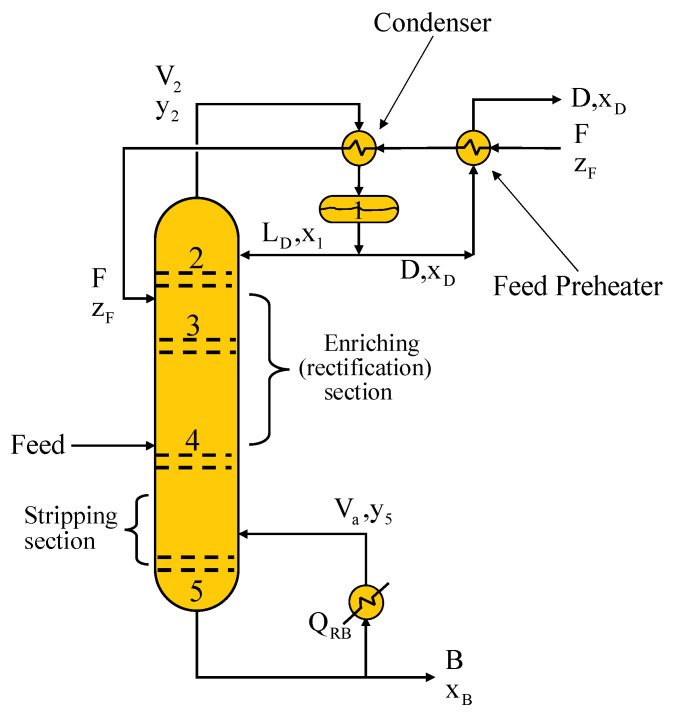
A distillation column.

Distillation is the basic operation in petrochemical and pharmaceutical processes [26]. Distillation is carried out in a vertical column whose chemical process consists of separating mixtures. This column contains plates used to enhance the component separations, a reboiler to provide heat for the necessary vaporization from the bottom of the column, a condenser to cool and condense the vapor from the top of the column and a reflux drum to hold the condensed vapor so that liquid reflux can be recycled back from the top of the column. A scheme of a distillation column is shown in Figure 1.

The liquid mixture that needs to be processed is known as the feed, which is entered near the middle of the column to a stage known as the feed tray. Considering the feed tray, the column is divided into the enriching or rectification section located at the top and the stripping section at the bottom. A reboiler located at the bottom collects the feed flow coming down from the column. Likewise, vapor is generated in the reboiler due to the supply of heat. The vapor raised in the reboiler is returned to the column at the bottom. The resulting liquid from the reboiler is called the bottom product. A condenser located at the top of the column is used to cool the vapor moving up the column and, in some integration schemes, as depicted in Figure 1, may be used to preheat the mixture to be fed to the column at the same time. The part of the recovered liquid in the condenser is stored in a holding vessel called a reflux drum. A quantity of the liquid is recycled back to the top of the column, and it is called reflux. Finally, the distillation is the condensed liquid removed from the system.

**Figure 2:**
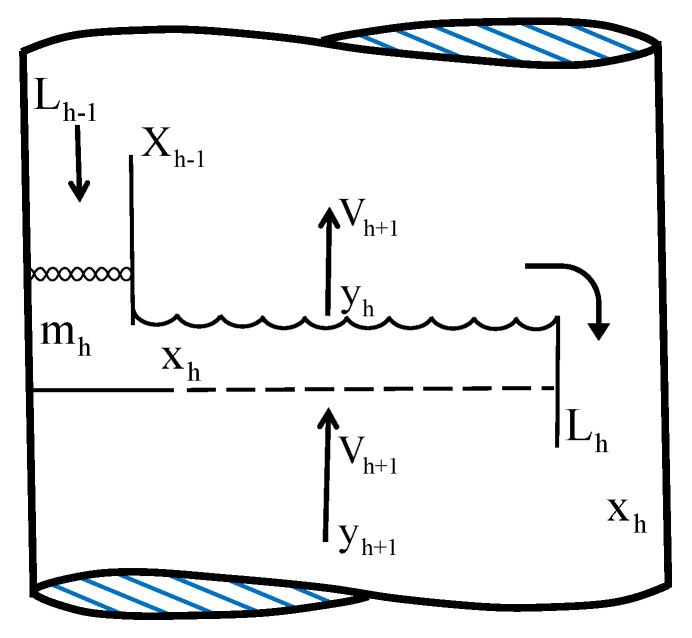
Section of a distillation column for a plate.

Figure 2 shows the direction of vapor and liquid flow across a tray and across a column.

Two conduits are installed at the ends of each tray called downcomers through which the liquid falls by gravity. Because the vapor is lighter, it flows up the column and contacts the liquid through openings in each tray. The area through which the vapor circulates in each tray is called the active tray area.

In order to find a mathematical model of the distillation column, the following assumptions are applied.

- Each stage is a perfectly mixed stage.
- The liquid and vapor leaving any stage are in physical equilibrium.
- The energy balance is based on the conservation of enthalpy instead of internal energy.
- There is no vapor retention at any stage.
- Flows and fractions are on a molar basis.
- The volume and number of moles in the liquid remain constant at each stage.
- Total moles of liquid in the condenser and reboiler is constant.

Consider a distillation column with nc components; in the feed stage, the overall mass balance is given by

(1) $$\frac{dn_{h}}{dt}=L_{h-1}+V_{h+1}+F-L_{h}-V_{h}$$

where $n_{h}$ are the total mole mass in the feed stage $\left(h\right),$ i.e., the total amount of matter in the stage expressed in moles, *F* is the molar flow rate for the supply and *V* and *L* are molar flows of vapor and liquid between stages, respectively.

The balance for each component *j* is expressed as

(2) $$\begin{array}{ccc} \frac{dn_{h,j}}{dt} & = & L_{h-1}x_{h-1,j}+V_{h+1}y_{h+1,j}+Fz_{F,j}-L_{h}x_{h,j}-V_{h}y_{h,j} \end{array}$$

(3) $$\begin{array}{ccc} \frac{dx_{h,j}m_{f}}{dt} & = & L_{h-1}x_{h-1,j}+V_{h+1}y_{h+1,j}+Fz_{F,j}-L_{h}x_{h,j}-V_{h}y_{h,j} \end{array}$$

where $n_{h}$ are the moles retained in the stage, $\frac{dn_{h,j}}{dt}$ is the term of accumulation, $n_{h,j}$ are the moles corresponding to component *j*, $\frac{dx_{h,j}m_{f}}{dt}$ accumulation in the stage, $x_{k,j}$ is the mole fraction of component *j* in the liquid leaving stage *k*, $y_{k,j}$ is the mole fraction of component *j* in the vapor leaving stage *k* and $z_{F,j}$ is the mole fraction of component *j* in the feed.

As the liquid retention in each state is constant,

(4) $$n_{h}\frac{dx_{h,j}}{dt}=L_{h-1}x_{h-1,j}+V_{h+1}y_{h+1,j}+Fz_{F,j}-L_{h}x_{h,j}-V_{h}y_{h,j}$$

also the flow of vapor and liquid between stages in the rectification and stripping sections is constant, for any stage *i* different from the feed stage,

(5) $$\begin{array}{ccc} V_{i} & = & V_{i+1}=V_{i-1} \end{array}$$

(6) $$\begin{array}{ccc} L_{i} & = & L_{i+1}=L_{i-1} \end{array}$$

The model expressed in mole fractions for a binary mixture, in terms of the most volatile component for a five-stage distillation column, is described by

(7) $$\begin{array}{ccc} \frac{dx_{1}}{dt} & = & -\frac{V_{R}}{m_{1}}x_{1}+\frac{V_{R}}{m_{1}}y_{2} \end{array}$$

(8) $$\begin{array}{ccc} \frac{dx_{2}}{dt} & = & \frac{L_{R}}{m_{2}}x_{1}-\frac{L_{R}}{m_{2}}x_{2}-\frac{V_{R}}{m_{2}}y_{2}+\frac{V_{R}}{m_{2}}y_{3} \end{array}$$

(9) $$\begin{array}{ccc} \frac{dx_{3}}{dt} & = & \frac{L_{R}}{m_{3}}x_{2}-\frac{L_{S}}{m_{3}}x_{3}-\frac{V_{R}}{m_{3}}y_{3}+\frac{V_{S}}{m_{3}}y_{4}+\frac{F}{m_{3}}z_{F} \end{array}$$

(10) $$\begin{array}{ccc} \frac{dx_{4}}{dt} & = & \frac{L_{S}}{m_{4}}x_{3}-\frac{L_{S}}{m_{4}}x_{4}-\frac{V_{S}}{m_{4}}y_{4}+\frac{V_{S}}{m_{4}}y_{5} \end{array}$$

(11) $$\begin{array}{ccc} \frac{dx_{5}}{dt} & = & \frac{L_{S}}{m_{5}}x_{4}-\frac{B}{m_{5}}x_{5}-\frac{V_{S}}{m_{5}}y_{5} \end{array}$$

where mole fraction (or composition) of the light component as a function on the composition of the same component in the liquid using the relative volatility formula α is expressed by

(12) $$y_{i}=\frac{\alpha x_{i}}{1+\left(\alpha -1\right)x_{i}}$$

The system described from (7) to (11) indicates

- $m_{1}$ is the number of moles of liquid in the condenser.
- $m_{2}$ to $m_{4}$ are the moles of liquid in the intermediate plates and, by design, equal to each other.
- $m_{5}$ are the moles in the reboiler.
- $x_{1}$ to $x_{5}$ are the mole fractions of the light component in the liquid from stages 1 to 5.
- $y_{1}$ to $y_{5}$ are the mole fractions of the light component in the vapor leaving stages 1 to 5.
- *F* is the molar feed flow to the column.
- $L_{S}$ and $L_{R}$ are the molar flows of liquid in the stripping and rectification section stages, respectively.
- $V_{S}$ and $V_{R}$ are the molar flows of vapor going up the stripping and rectification, respectively.
- *B* is the molar flow of bottom product leaving the column.

The basic elements of bond graph modeling of systems are described in the next section.

## 3. Bond Graph Models

**Figure 3:**
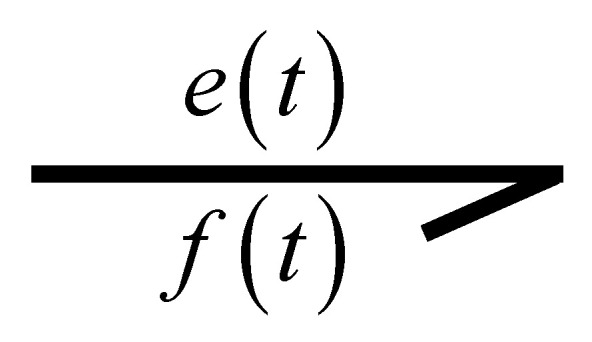
Power bond.

Power transfer is an essential property in bond graph modeling of systems. Using generalized variables, power is defined as the product of effort $e\left(t\right)$ and flow $f\left(t\right)$. Power transmission from one port to another port is drawn by a simple line with direction, which is called the power bond and is shown in Figure 3 [21].

Generalized power variables in different energy domains are indicated in Table 1.

**Figure 4:**
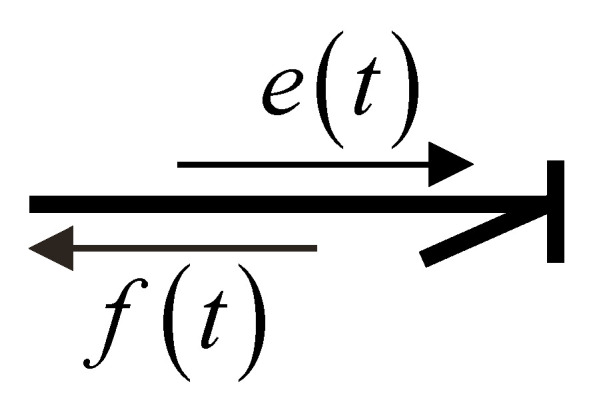
Causal power bond.

Additionally, two energy variables are used in this graphical modeling called moment $p\left(t\right)$ and displacement $q\left(t\right)$ and are related to the power variables by $p\left(t\right)=\int e\left(t\right)dt$ and $q\left(t\right)=\int f\left(t\right)dt$. The relationships of the variables in a bond graph are determined by causality, which is applied to each bond by a vertical stroke, as illustrated in Figure 4 [21].

**Figure 5:**
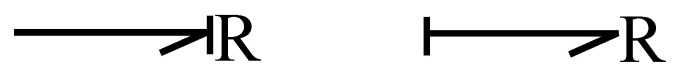
Resistor.

The elements that are part of a model in the bond graph are the following:

- Passive elements store or dissipate energy. Resistors are characterized by being dissipative elements, as shown in Figure 5.

The causal relationship defines

(13) $$\begin{array}{ccc} e\left(t\right) & = & \Phi_{R}\left[f\left(t\right)\right] \end{array}$$

(14) $$\begin{array}{ccc} f\left(t\right) & = & \Phi_{R}^{-1}\left[e\left(t\right)\right] \end{array}$$

**Figure 6:**
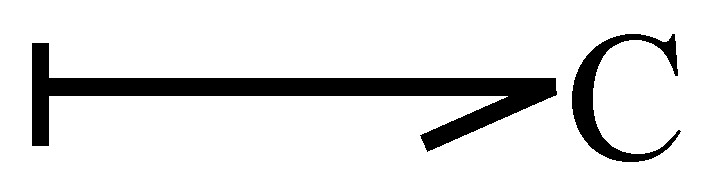
Capacitance in integral causality.

**Figure 7:**
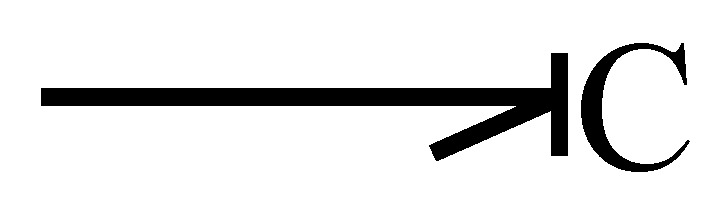
Capacitance in derivative causality.

The elements that store energy are inertia or capacitance. Figure 6 illustrates a capacitance in integral causality assigned, and the relationship of its variables is expressed by

(15) $$e\left(t\right)=\phi_{C}^{-1}\left[\int f\left(t\right)dt\right]$$

when this element is in derivative causality it is defined by

(16) $$f\left(t\right)=\frac{d\phi_{C}\left[e\left(t\right)\right]}{dt}$$

and is shown in Figure 7.

**Figure 8:**
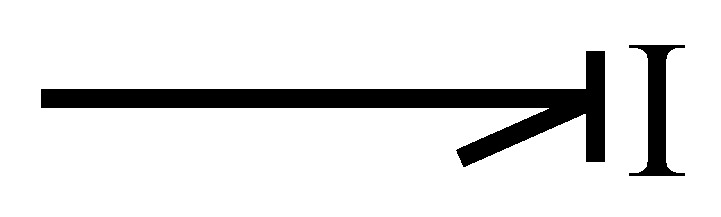
Inertia in integral causality.

**Figure 9:**
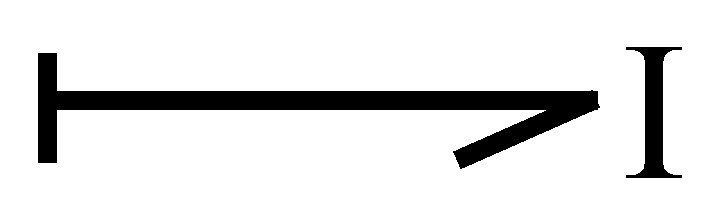
Inertia in derivative causality.

Inertia is the other energy storage element, and its representation in integral causality is illustrated in Figure 8.

whose causal relationship is given by

(17) $$f\left(t\right)=\phi_{I}^{-1}\left[\int e\left(t\right)dt\right]$$

this element in derivative causality is defined by

(18) $$e\left(t\right)=\frac{d\phi_{I}\left[f\left(t\right)\right]}{dt}$$

and is shown in Figure 9.

**Figure 10:**
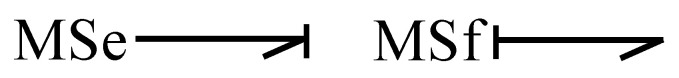
Active elements: sources of effort and flow.

**Figure 11:**
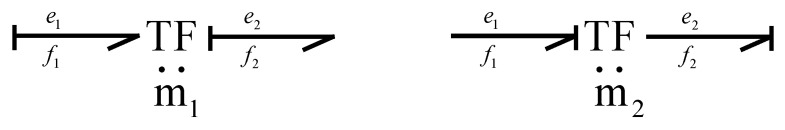
Transformers.

- Active elements are representative elements of sources that supply power to the system due to the two variables. There are two sources that are shown in Figure 10.

- Transformation elements change the magnitude or type of signal to another. One of these elements is transformers, as shown in Figure 11.

Causal relationships are described by

(19) $$\begin{array}{ccc} \left[\begin{array}{c} e_{1}\left(t\right)\\ f_{1}\left(t\right) \end{array}\right] & = & \left[\begin{array}{cc} m_{1} & 0\\ 0 & \frac{1}{m_{1}} \end{array}\right]\left[\begin{array}{c} e_{2}\left(t\right)\\ f_{2}\left(t\right) \end{array}\right] \end{array}$$

(20) $$\begin{array}{ccc} \left[\begin{array}{c} e_{2}\left(t\right)\\ f_{2}\left(t\right) \end{array}\right] & = & \left[\begin{array}{cc} m_{2} & 0\\ 0 & \frac{1}{m_{2}} \end{array}\right]\left[\begin{array}{c} e_{1}\left(t\right)\\ f_{1}\left(t\right) \end{array}\right] \end{array}$$

depending on the causality.

**Figure 12:**
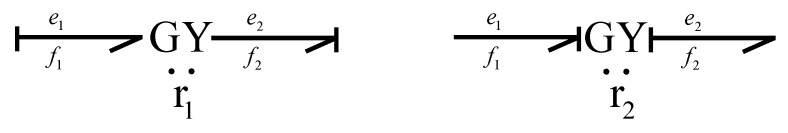
Gyrators.

Figure 12 shows the element that changes the type of the signal

and according to the applied causality, the expressions are given by

(21) $$\begin{array}{ccc} \left[\begin{array}{c} e_{1}\left(t\right)\\ f_{1}\left(t\right) \end{array}\right] & = & \left[\begin{array}{cc} 0 & r_{1}\\ \frac{1}{r_{1}} & 0 \end{array}\right]\left[\begin{array}{c} e_{2}\left(t\right)\\ f_{2}\left(t\right) \end{array}\right] \end{array}$$

(22) $$\begin{array}{ccc} \left[\begin{array}{c} e_{2}\left(t\right)\\ f_{2}\left(t\right) \end{array}\right] & = & \left[\begin{array}{cc} 0 & r_{2}\\ \frac{1}{r_{2}} & 0 \end{array}\right]\left[\begin{array}{c} e_{1}\left(t\right)\\ f_{1}\left(t\right) \end{array}\right] \end{array}$$

**Figure 13:**
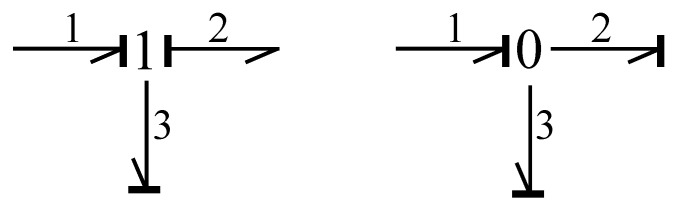
Junctions.

- Connection elements are defined by the series junction (1) and the parallel junction (0), and through these junctions, the connection of the bonds is made. Figure 13 illustrates these junctions.

According to Figure 10, junction 1 determines the following expressions for efforts and flows,

(23) $$f_{1}\left(t\right)=f_{2}\left(t\right)=f_{3}\left(t\right);\;e_{1}\left(t\right)-e_{2}\left(t\right)-e_{3}\left(t\right)=0$$

and for junction 0, they are

(24) $$f_{1}\left(t\right)-f_{2}\left(t\right)-f_{3}\left(t\right)=0;\;e_{1}\left(t\right)=e_{2}\left(t\right)=e_{3}\left(t\right)$$

**Figure 14:**
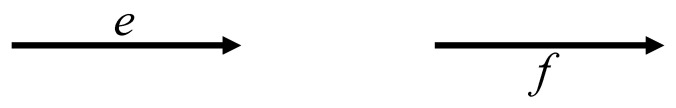
Active bond.

- Active bonds are used as information bonds and are drawn by full arrows indicating the signal transmitted by a sensor, a summation element or, in general, one of the generalized power variables, as shown in Figure 14.

## 4. Junction Structure of Bond Graph Models for a Class of Nonlinear Systems

Bond graph modeling of systems has been characterized by determining structural properties [30]. The assignment of causality to the different elements of a bond graph allows to directly find structural controllability and structural observability, steady-state response and linearization [23]. One of the keys to carry out the formal mathematical proof of these analyses is to obtain the junction structure of the bond graph.

The junction structure classifies the different elements of the model into fields. Hence, in this paper, a junction structure that allows the modeling of a distillation column in a bond graph is proposed. This structure is based on the fields of sources and detectors that determine inputs and outputs, respectively, the storage field that has elements in integral and derivative causality assigning linearly independent and dependent state variables, respectively, and the field of dissipative elements has been divided into linear and nonlinear elements and can also be modulated.

**Figure 15:**
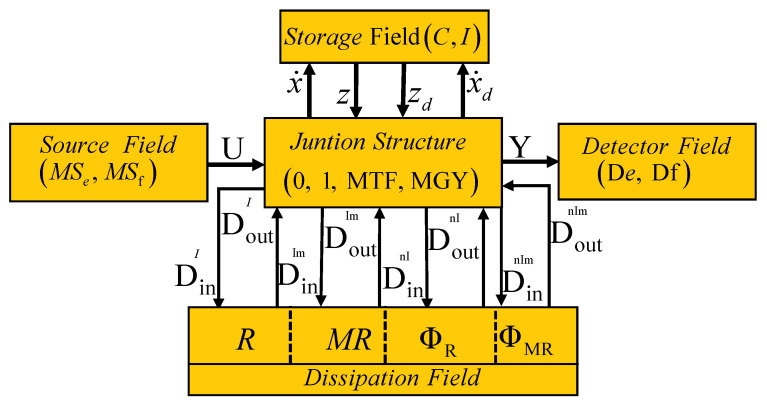
Junction structure for nonlinear systems modeled by bond graphs.

The proposed junction structure is shown in Figure 15 with the following elements:

- System inputs $u\left(t\right)\in {ℜ}^{p}$ through the effort and flow sources denoted by $MS_{e}$ and $MS_{f}$, respectively.
- System outputs $y\left(t\right)\in {ℜ}^{q}$ through effort and flow detectors denoted by $D_{e}$ and $D_{f}$, respectively.
- Linearly independent state variables $x\left(t\right)\in {ℜ}^{n}$ are storage elements in integral causality *C* or *I* with power variables $\overset{•}{x}\left(t\right)\in {ℜ}^{n}$ and $z\left(t\right)\in {ℜ}^{n}$.
- Linearly dependent state variables $x_{d}\left(t\right)\in {ℜ}^{m}$ are storage elements in derivative causality *C* or *I* with power variables ${\overset{•}{x}}_{d}\left(t\right)\in {ℜ}^{m}$ and $z_{d}\left(t\right)\in {ℜ}^{m}$.
- Linear and nonlinear algebraic relationships through the dissipative field are divided into:
  — Linear dissipation elements denoted by *R* with key vectors $D_{in}^{l}\in {ℜ}^{r_{l}}$ and $D_{out}^{l}\in {ℜ}^{r_{l}}$.
  — Modulated linear dissipation elements denoted by MR with key vectors $D_{in}^{lm}\in {ℜ}^{r_{lm}}$ and $D_{out}^{lm}\in {ℜ}^{r_{lm}}$.
  — Nonlinear dissipation elements denoted by $\phi_{R}$ with key vectors $D_{in}^{nl}\in {ℜ}^{r_{nl}}$ and $D_{out}^{nl}\in {ℜ}^{r_{nl}}$.
  — Modulated nonlinear dissipation elements denoted by $\phi_{MR}$ with key vectors $D_{in}^{nlm}\in {ℜ}^{r_{nlm}}$ and $D_{out}^{nlm}\in {ℜ}^{r_{nlm}}$.
- The junction structure formed by
  — Junctions 1 and 0 that determine the connection between the elements.
  — Transformers and gyrators denoted by MTF and MGY, respectively.

The relationships between inputs and outputs of the junction structure of Figure 15 determine the mathematical model of the bond graph model. Due to the fact that this structure allows modulated elements in general, the model represents a class of nonlinear systems, and through the following lemma, the formal relationships of the proposed junction structure and its representation in state space are established.

**Lemma** **1.** *Consider a physical system modeled by a bond graph model in a predefined integral causality assignment whose block diagrams, key vectors and interconnections are illustrated in Figure 15. The junction structure is defined by*

(25) $$\left[\begin{array}{c} \overset{•}{x}\left(t\right)\\ D_{in}^{l}\left(t\right)\\ D_{in}^{lm}\left(t\right)\\ D_{in}^{nl}\left(t\right)\\ D_{in}^{nlm}\left(t\right)\\ y\left(t\right)\\ z_{d}\left(t\right) \end{array}\right]=\left[\begin{array}{ccccccc} S_{11}\left(x\right) & S_{12}^{11}\left(x\right) & S_{12}^{12}\left(x\right) & S_{12}^{13}\left(x\right) & S_{12}^{14}\left(x\right) & S_{13}\left(x\right) & S_{14}\left(x\right)\\ S_{21}^{11}\left(x\right) & S_{22}^{11}\left(x\right) & S_{22}^{12}\left(x\right) & 0 & 0 & S_{23}^{11}\left(x\right) & 0\\ S_{21}^{21}\left(x\right) & S_{22}^{21}\left(x\right) & S_{22}^{22}\left(x\right) & 0 & 0 & S_{23}^{21}\left(x\right) & 0\\ S_{21}^{31}\left(x\right) & 0 & 0 & 0 & 0 & S_{23}^{31}\left(x\right) & 0\\ S_{21}^{41}\left(x\right) & 0 & 0 & 0 & 0 & S_{23}^{41}\left(x\right) & 0\\ S_{31}\left(x\right) & S_{32}^{11}\left(x\right) & S_{32}^{12}\left(x\right) & S_{32}^{13}\left(x\right) & S_{32}^{14}\left(x\right) & S_{33}\left(x\right) & 0\\ S_{41}\left(x\right) & 0 & 0 & 0 & 0 & 0 & 0 \end{array}\right]\left[\begin{array}{c} z\left(t\right)\\ D_{out}^{l}\left(t\right)\\ D_{out}^{lm}\left(t\right)\\ D_{out}^{nl}\left(t\right)\\ D_{out}^{nlm}\left(t\right)\\ u\left(t\right)\\ {\overset{•}{x}}_{d}\left(t\right) \end{array}\right]$$

*with entries of $S\left(x\right)$ inside the set $\left\{0,\;\pm 1,\;\pm f_{T}\left(x\right),\;\pm f_{G}\left(x\right)\right\},$ where $f_{T}\left(x\right)$ and $f_{G}\left(x\right)$ are state functions that modulate MTF and MGY, respectively; and the constitutive relations of the fields are given by*

(26) $$\begin{array}{ccc} z\left(t\right) & = & Fx\left(t\right) \end{array}$$

(27) $$\begin{array}{ccc} z_{d}\left(t\right) & = & F_{d}x_{d}\left(t\right) \end{array}$$

(28) $$\begin{array}{ccc} D_{out}^{l}\left(t\right) & = & LD_{in}^{l}\left(t\right) \end{array}$$

(29) $$\begin{array}{ccc} D_{out}^{lm}\left(t\right) & = & L_{m}D_{in}^{lm}\left(t\right) \end{array}$$

(30) $$\begin{array}{ccc} D_{out}^{nl}\left(t\right) & = & \phi \left(D_{in}^{nl}\left(t\right)\right) \end{array}$$

(31) $$\begin{array}{ccc} D_{out}^{nlm}\left(t\right) & = & \phi_{m}\left(D_{in}^{nlm}\left(t\right)\right) \end{array}$$

*then a state variable representation for this class of nonlinear systems is described by*

(32) $$\overset{•}{x}\left(t\right)=A\left(x\right)x\left(t\right)+B\left(x\right)u\left(t\right)+H\left(x,u\right)$$

*where*

$$\begin{array}{ccc} A\left(x\right) & = & E^{-1}\left(x\right)\left[S_{11}^{11}\left(x\right)+S_{12}^{11}\left(x\right)Q\left(x\right)S_{A}\left(x\right)+\right. \end{array}$$

(33) $$\begin{array}{cccc} & & & \left.S_{12}^{12}\left(x\right)Q_{m}\left(x\right)S_{Am}\left(x\right)+S_{14}\left(x\right)F_{d}^{-1}{\overset{•}{S}}_{14}\left(x\right)\right]F \end{array}$$

(34) $$\begin{array}{ccc} B\left(x\right) & = & E^{-1}\left(x\right)\left[S_{13}\left(x\right)+S_{12}^{11}\left(x\right)Q\left(x\right)S_{B}\left(x\right)\right] \end{array}$$

(35) $$\begin{array}{ccc} H\left(x,u\right) & = & E^{-1}\left(x\right)\left[S_{21}^{13}\left(x\right)D_{out}^{nl}\left(t\right)+S_{12}^{14}\left(x\right)D_{out}^{nlm}\left(t\right)\right] \end{array}$$

(36) $$\begin{array}{ccc} E\left(x\right) & = & I-S_{14}\left(x\right)F_{d}^{-1}S_{41}\left(x\right)F \end{array}$$

*with*

(37) $$\begin{array}{ccc} S_{A}\left(x\right) & = & S_{21}^{11}\left(x\right)+S_{22}^{12}\left(x\right)M_{m}\left(x\right)S_{21}^{21}\left(x\right) \end{array}$$

(38) $$\begin{array}{ccc} S_{Am}\left(x\right) & = & S_{21}^{21}\left(x\right)+S_{22}^{21}\left(x\right)M\left(x\right)S_{21}^{11}\left(x\right) \end{array}$$

(39) $$\begin{array}{ccc} S_{B}\left(x\right) & = & S_{23}^{11}\left(x\right)+S_{22}^{12}\left(x\right)M_{m}\left(x\right)S_{23}^{21}\left(x\right) \end{array}$$

(40) $$\begin{array}{ccc} S_{Bm}\left(x\right) & = & S_{23}^{21}\left(x\right)+S_{22}^{21}\left(x\right)M\left(x\right)S_{23}^{11}\left(x\right) \end{array}$$

*and*

(41) $$\begin{array}{ccc} Q\left(x\right) & = & L{\left[I-S_{22}^{11}\left(x\right)L-S_{22}^{12}\left(x\right)M_{m}\left(x\right)S_{22}^{21}\left(x\right)L\right]}^{-1} \end{array}$$

(42) $$\begin{array}{ccc} Q_{m}\left(x\right) & = & L_{m}{\left[I-S_{22}^{22}\left(x\right)L_{m}-S_{22}^{21}\left(x\right)M\left(x\right)S_{22}^{12}\left(x\right)L_{m}\right]}^{-1} \end{array}$$

(43) $$\begin{array}{ccc} M\left(x\right) & = & L{\left[I-S_{22}^{11}\left(x\right)L\right]}^{-1} \end{array}$$

(44) $$\begin{array}{ccc} M_{m}\left(x\right) & = & L_{m}{\left[I-S_{22}^{22}\left(x\right)L_{m}\right]}^{-1} \end{array}$$

The proof of Lemma 1 is given in Appendix A.

The proposed methodology to obtain a distillation column model in a bond graph approach is applied in the next section.

## 5. Bond Graph Model of a Distillation Column

A distillation column is a multidomain energy system. However, for the purposes of this paper, the variables of interest for the column are described in Section 2.

**Figure 16:**
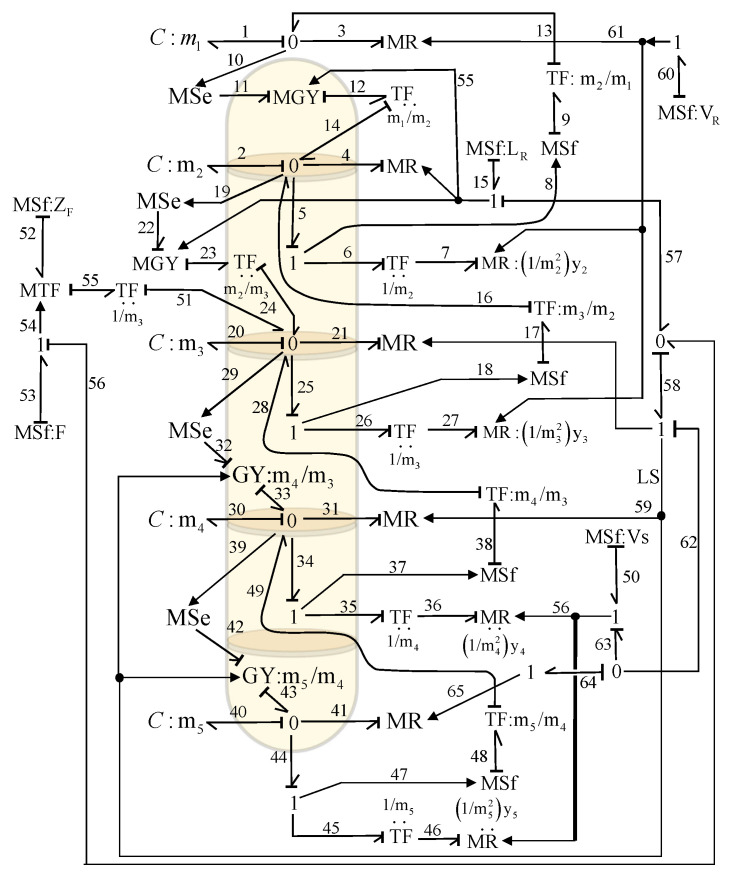
Bond Graph for a Distillation Column.

In the bond graph approach, this column represents a chemical system whose power variables are: chemical potential and molar flow as effort and flow, respectively. The column has five stages that determine five different chemical tensions, and the bond graph will have five 0-junctions. Storage elements *C* are connected to each of these 0-junctions, resulting in the accumulation of components. From each stage, there is a molar flow of the component leaving that stage in the vapor phase $V_{R}y_{i}=V_{i,1}$. These terms are modeled as dissipative elements *R*, and the molar flow of the light component in the liquid phase leaving stage *i* indicates the connection of another element *R*. In this way, the elements are connected, and each 0-junction expresses the equation of each plate of the column. Therefore, a bond graph model of a distillation column of five plates is illustrated in Figure 16.

The basic elements of the distillation column bond graph are:

- Five major 0-junctions of each chemical tension corresponding to each plate of the column.
- Five storage elements *C* of each flow accumulator for each tray.
- MR elements are modulated by molar flows and considering the relative volatility.
- Transformers TF and gyrators MGY are used to couple the different input and output flows to each tray.

In order to obtain the mathematical model of a bond graph model, the variables of the different fields, according to Figure 15, are obtained. Hence, the key vectors for the storage elements are expressed by

(45) $$x\left(t\right)=\left[\begin{array}{c} q_{1}\\ q_{2}\\ q_{20}\\ q_{30}\\ q_{40} \end{array}\right];\overset{•}{x}\left(t\right)=\left[\begin{array}{c} f_{1}\\ f_{2}\\ f_{20}\\ f_{30}\\ f_{40} \end{array}\right];z\left(t\right)=\left[\begin{array}{c} e_{1}\\ e_{2}\\ e_{20}\\ e_{30}\\ e_{40} \end{array}\right]$$

the decomposition of the dissipation field and its key vectors are described by

(46) $$D_{in}^{lm}\left(t\right)=\left[\begin{array}{c} e_{4}\\ e_{3}\\ e_{21}\\ e_{31}\\ e_{41} \end{array}\right];D_{out}^{lm}\left(t\right)=\left[\begin{array}{c} f_{4}\\ f_{3}\\ f_{21}\\ f_{31}\\ f_{41} \end{array}\right];D_{in}^{nlm}\left(t\right)=\left[\begin{array}{c} e_{7}\\ e_{27}\\ e_{36}\\ e_{46} \end{array}\right];D_{out}^{nlm}\left(t\right)=\left[\begin{array}{c} f_{7}\\ f_{27}\\ f_{36}\\ f_{46} \end{array}\right]$$

with the input

(47) $$u\left(t\right)=f_{53}$$

The linear constitutive relationships are given by

(48) $$\begin{array}{ccc} F & = & diag\left\{\frac{1}{m_{1}},\frac{1}{m_{2}},\frac{1}{m_{3}},\frac{1}{m_{4}},\frac{1}{m_{5}}\right\} \end{array}$$

(49) $$\begin{array}{ccc} L_{m} & = & diag\left\{L_{R},V_{R},L_{S},L_{S},B\right\} \end{array}$$

and nonlinear as

(50) $$\left[\begin{array}{c} f_{7}\\ f_{27}\\ f_{36}\\ f_{46} \end{array}\right]=\left[\begin{array}{c} \frac{V_{R}}{m_{2}^{2}}y\left(e_{7}\right)\\ \frac{V_{R}}{m_{3}^{2}}y\left(e_{27}\right)\\ \frac{V_{S}}{m_{4}^{2}}y\left(e_{36}\right)\\ \frac{V_{S}}{m_{5}^{2}}y\left(e_{46}\right) \end{array}\right]$$

where

(51) $$\begin{array}{ccc} y\left(e_{7}\right) & = & \frac{\alpha e_{7}}{1+\left(\alpha -1\right)e_{7}} \end{array}$$

(52) $$\begin{array}{ccc} y\left(e_{27}\right) & = & \frac{\alpha e_{27}}{1+\left(\alpha -1\right)e_{27}} \end{array}$$

(53) $$\begin{array}{ccc} y\left(e_{36}\right) & = & \frac{\alpha e_{36}}{1+\left(\alpha -1\right)e_{36}} \end{array}$$

(54) $$\begin{array}{ccc} y\left(e_{46}\right) & = & \frac{\alpha e_{46}}{1+\left(\alpha -1\right)e_{46}} \end{array}$$

The junction structure of the bond graph is defined by

(55) f1f2f20f30f40e4e3e21e31e41e7e27e36e46=000000−1000m22m10000m1m2LR0000−10000−m2m32m20000m2m3LR00000−1000−m3m42m30f52m300m3m4LS00000−1000−m4m52m40000m4m5LS00000−1000−m500100000000000001000000000000000010000000000000001000000000000000100000000000m2000000000000000m3000000000000000m4000000000000000m50000000000e1e2e20e30e40f4f3f21f31f41f7f27f36f46f53

There are no storage elements in derivative causality assignment in the bond graph model, so there are no linearly dependent variables, and the matrix $E\left(x\right)=I$. For this case, since $S_{22}^{11}\left(x\right)=0$, $S_{22}^{22}\left(x\right)=0$, then $M\left(x\right)=L$, $M_{m}\left(x\right)=L_{m}T$, $Q\left(x\right)=L$ and $Q_{m}\left(x\right)=L_{m}$. From (33), (37), (38) with (48), (49) and (55),

(56) $$A\left(x\right)=\left[\begin{array}{ccccc} \frac{-V_{R}}{m_{1}} & 0 & 0 & 0 & 0\\ \frac{L_{R}}{m_{2}} & \frac{-L_{R}}{m_{2}} & 0 & 0 & 0\\ 0 & \frac{L_{R}}{m_{3}} & \frac{-L_{S}}{m_{3}} & 0 & 0\\ 0 & 0 & \frac{L_{S}}{m_{4}} & \frac{-L_{S}}{m_{4}} & 0\\ 0 & 0 & 0 & \frac{L_{S}}{m_{5}} & \frac{-B}{m_{5}} \end{array}\right]$$

From (34), (39), (40) with (55),

(57) $$B\left(x\right)=\left[\begin{array}{c} 0\\ 0\\ \frac{f_{52}}{m_{3}}\\ 0\\ 0 \end{array}\right]$$

From (35) with (50) and (55),

(58) $$H\left(x,u\right)=\left[\begin{array}{c} \frac{V_{R}}{m_{1}}y\left(e_{7}\right)\\ \frac{-V_{R}}{m_{2}}y\left(e_{7}\right)+\frac{V_{R}}{m_{2}}y\left(e_{27}\right)\\ \frac{-V_{R}}{m_{3}}y\left(e_{27}\right)+\frac{V_{R}}{m_{1}}y\left(e_{36}\right)\\ \frac{-V_{S}}{m_{4}}y\left(e_{36}\right)+\frac{V_{S}}{m_{4}}y\left(e_{46}\right)\\ \frac{-V_{S}}{m_{5}}y\left(e_{46}\right) \end{array}\right]$$

The state space of the distillation column from (56), (57) and (58) is defined by

(59) $$\overset{•}{x}\left(t\right)=\left[\begin{array}{ccccc} \frac{-V_{R}}{m_{1}} & 0 & 0 & 0 & 0\\ \frac{L_{R}}{m_{2}} & \frac{-L_{R}}{m_{2}} & 0 & 0 & 0\\ 0 & \frac{L_{R}}{m_{3}} & \frac{-L_{S}}{m_{3}} & 0 & 0\\ 0 & 0 & \frac{L_{S}}{m_{4}} & \frac{-L_{S}}{m_{4}} & 0\\ 0 & 0 & 0 & \frac{L_{S}}{m_{5}} & \frac{-B}{m_{5}} \end{array}\right]x\left(t\right)+\left[\begin{array}{c} \frac{V_{R}}{m_{1}}y\left(e_{7}\right)\\ \frac{-V_{R}}{m_{2}}y\left(e_{7}\right)+\frac{V_{R}}{m_{2}}y\left(e_{27}\right)\\ \frac{-V_{R}}{m_{3}}y\left(e_{27}\right)+\frac{V_{R}}{m_{1}}y\left(e_{36}\right)\\ \frac{-V_{S}}{m_{4}}y\left(e_{36}\right)+\frac{V_{S}}{m_{4}}y\left(e_{46}\right)\\ \frac{-V_{S}}{m_{5}}y\left(e_{46}\right) \end{array}\right]+\left[\begin{array}{c} 0\\ 0\\ \frac{f_{52}}{m_{3}}\\ 0\\ 0 \end{array}\right]u\left(t\right)$$

in a developed form with (46), the distillation column has the form

(60) $$\begin{array}{ccc} m_{1}\frac{dq_{1}}{dt} & = & -V_{R}q_{1}+V_{R}y\left(q_{2}\right) \end{array}$$

(61) $$\begin{array}{ccc} m_{2}\frac{dq_{2}}{dt} & = & L_{R}q_{1}-L_{R}q_{2}-V_{R}y\left(q_{2}\right)+V_{R}y\left(q_{3}\right) \end{array}$$

(62) $$\begin{array}{ccc} m_{3}\frac{dq_{3}}{dt} & = & L_{R}q_{2}-L_{S}q_{3}-V_{R}y\left(q_{3}\right)+V_{S}y\left(q_{4}\right)+f_{52}\cdot u \end{array}$$

(63) $$\begin{array}{ccc} m_{4}\frac{dq_{4}}{dt} & = & L_{S}q_{3}-L_{S}q_{4}-V_{S}y\left(q_{4}\right)+V_{S}y\left(q_{5}\right) \end{array}$$

(64) $$\begin{array}{ccc} m_{5}\frac{dq_{5}}{dt} & = & L_{S}q_{4}-Bq_{5}-V_{S}y\left(q_{5}\right) \end{array}$$

Comparing the equations from (7) to (11) with respect to (60) to (64), it is concluded that the model built in the bond graph of the distillation column effectively represents the mathematical model of this system.

## 6. Distillation Columns Simulation in the Physical Domain

A generic design distillation column adapted to the conditions of the case studies is used. The architecture of the system is of a column designed for crude oil with bubble plates and a total condenser modified for distillation of fatty acids under vacuum.

The difference in the volatilities of the components of a mixture determines the degree of difficulty of the separation and, to a large extent, the complexity of the equipment to be used. This difference is evaluated by means of the relative volatility α, which is the ratio of the volatility of the most volatile component (light) over that of the least volatile (heavy). The greater its value, the easier the separation and the lower the number of steps required for a given separation, and decreasing α increases the number of stages in the column.

Two case studies with different relative volatility based on a distillation column bond graph were simulated. The 20 Sim software was used to perform the simulations in a bond graph environment.

### 6.1. First Case Study

The distillation column that is proposed to be simulated has a binary mixture with a low relative volatility. The nominal operating conditions of the column are described in Table 2. These conditions are based on classic cases from the literature [27,28,29], adapted and scaled into five stages.

The input variables to the system are defined by $L_{R}$ (distillate reflux to column dome),$V_{S}$ (bottom reflux to the reboiler), $z_{F}$ (mole fraction of light component in the feed) and *F* (molar feed flow) being the initial conditions given in Table 3.

The simulation results are obtained from increments of 10% of its supplied value, so the input vector with these variations is expressed by

(65) $${\left[\begin{array}{c} L_{R}\\ V_{S}\\ z_{F}\\ F \end{array}\right]}_{\delta }=\left[\begin{array}{c} 0.1*L_{R}\\ 0.1*V_{S}\\ 0.1*z_{F}\\ 0.1*F \end{array}\right]$$

and the activation times for the given changes are described by

(66) $${\left[\begin{array}{c} t_{L_{R}}\\ t_{V_{S}}\\ t_{z_{F}}\\ t_{F} \end{array}\right]}_{\delta }=\left[\begin{array}{c} 90\\ 140\\ 50\\ 10 \end{array}\right]\mathrm{minutes}$$

These time periods are calculated by the time required for the stabilization of the light component and the activation sequence of the different inputs. The sequence of input changes was selected to first introduce the changes that can be caused by external variations (feed flow and composition) and then those that can be used with the greatest effect in controlling the outputs.

The critical variables in the process are the concentrations of the light component in both the distillate and bottom flows since they are the ones that determine the degree of separation achieved.

**Figure 17:**
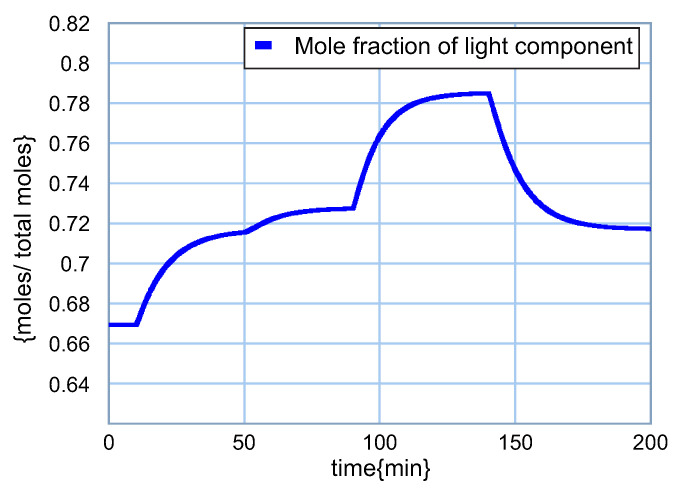
Behavior of the light component $q_{1}$.

The dynamic behavior of the light component $q_{1}$ is illustrated in Figure 17. After 10 min of steady-state operation, the feed flow is increased by 10%, so the magnitude of the light component also increases. According to the graph, the concentration of the light component in the distillate increases from $q_{1}=0.6693$ at t=0 to $q_{1}=0.7154$ at t=50 min, which is an increase of 6.9% with a 10% change in input. The next change is the feed composition, which also increases the light component to $q_{1}=0.7274$ at t=90 min, representing an increase of 1.7% at a 10% input change.

The increase in the return of the distillate to the column at t=90 min produces an increase in the mole fraction of light in the distillate to $q_{1}=0.7850$ at t=140 min, which determines a 7.9% increase due to a 10% increase in this input. Finally, the increase in bottom reflow produces a decrease in the concentration of the light component throughout the separation stages and consequently also in the distillate. In this way, a 10% increase in the return to the reboiler produces a change to $q_{1}=0.7172$, which determines a decrease of 8.6%, representing the inverse effect with respect to the previous changes.

**Figure 18:**
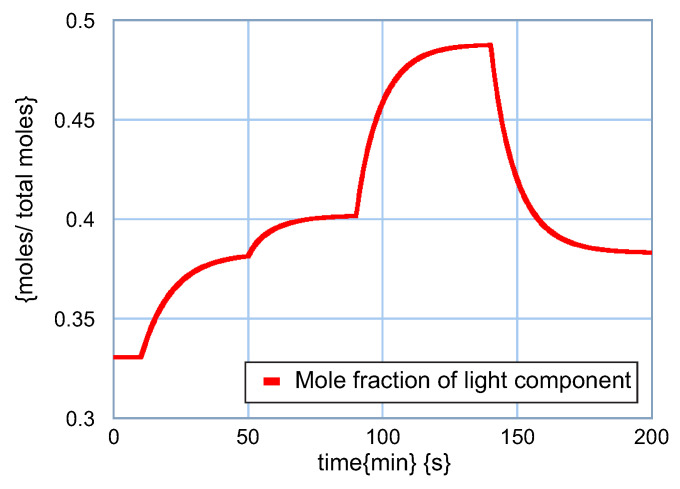
Concentration light in the bottom $q_{5}$.

Using the changes in the system inputs given by 65 and subject to the distribution of time intervals defined by (66), the dynamic behavior in the concentration of light in the bottom $q_{5}$ is shown in Figure 18.

According to Figure 18, the concentrations are lower; however, it is clearly seen that the evolution of the changes is similar to $q_{1}$.

**Figure 19:**
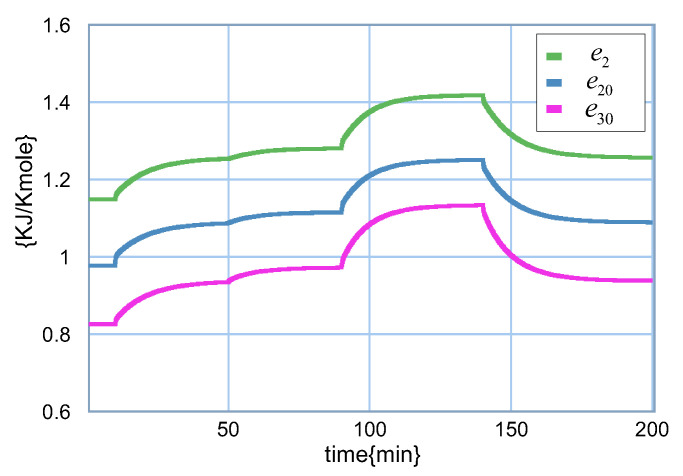
Chemical potential of stages 3, 4 and 5.

An interesting aspect of the bond graph model of the distillation column is that the chemical potential can be obtained. Therefore, the behavior of the chemical potential of stages 3, 4 and 5 are shown in Figure 19.

### 6.2. Second Case Study

The distillation column considered in this case has a binary mixture with a high relative volatility that allows easy separation of the mixture without requiring many column stages. In this case, the nominal data of the column are indicated in Table 4 [27,28,29].

The steady-state values of the system are described by Table 5.

The inputs to the system are the same as in the previous case, with a variation of increments of 10% of their nominal values defined by

$${\left[\begin{array}{c} L_{R}\\ V_{S}\\ z_{F}\\ F \end{array}\right]}_{\delta }=\left[\begin{array}{c} 0.9776\\ 1.5886\\ 0.6\\ 1 \end{array}\right]$$

the activation times for each of the inputs are defined by (66).

**Figure 20:**
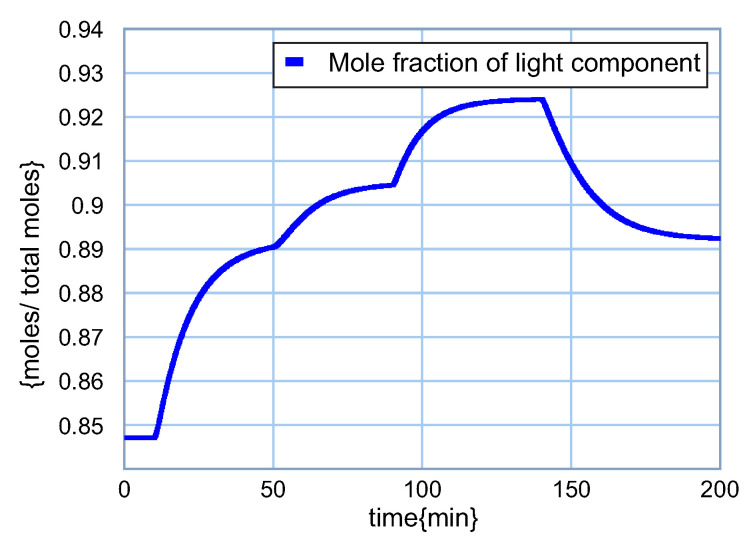
Response of the light component in the distillate.

The performance of the light component in the distillate $q_{1}$ is illustrated in Figure 20.

According to Figure 20, in the range of variation from 10 to 50 min, the feed is richer in the light component and introduces a greater amount of light component in the distillate, increasing from $q_{1}=0.8472$ to $q_{2}=0.8903$ at t=50 min with a 5.1% increase due to a 10% change in that input. The feed composition is then increased, and the mole fraction of light in the distillate increases to $q_{1}=0.9043$ at t=90 min, representing an increase of 1.6% with a 10% increase in this input. With the 10% increase in distillate return, the mole fraction of the lighter in the distillate also increases to $q_{1}=0.9240$ at t=140 min, indicating a percentage increase of 2.2%. Finally, a 10% increase in the bottom reflow produces a decrease in the concentration of the light component throughout the separation stages and, consequently, also in the distillate to 0.8924 at t=200 min, which determines a reduction in percentage to 3.4%.

**Figure 21:**
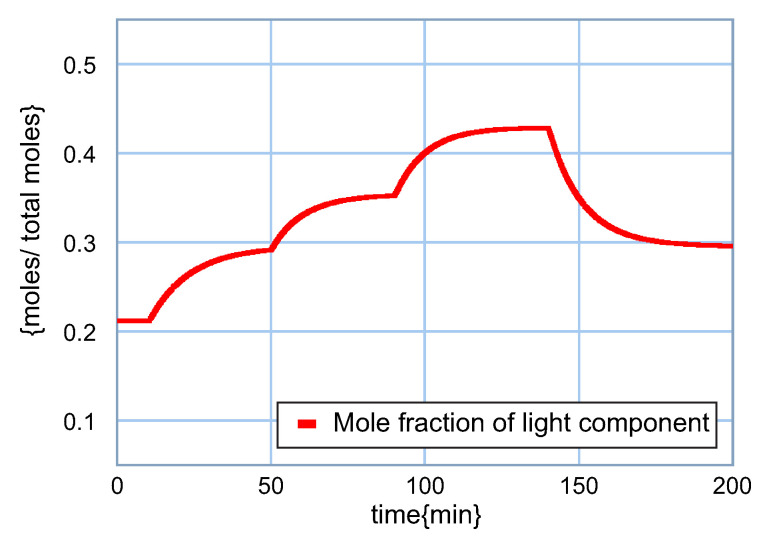
Response of the light component in the bottom.

The behavior in the composition of the flow of bottom shown in Figure 21 has a similar performance to the composition of the light component; however, the concentrations of light have much lower magnitudes as expected.

**Figure 22:**
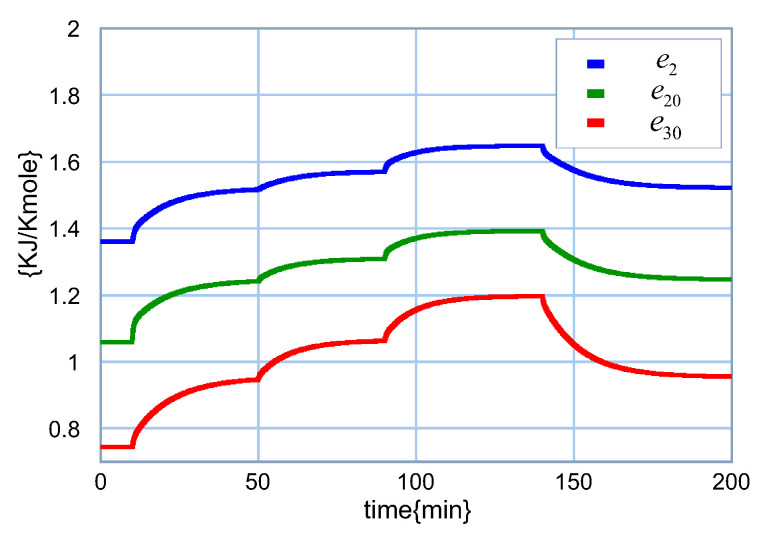
Chemical tension of stages 3, 4 and 5.

The performance of chemical potentials of stages 3, 4 and 5 are illustrated in Figure 22.

The response of the chemical tensions have forms similar to the concentration of the light component because it depends directly on this concentration. From the bond graph point of view, the measurement of these chemical tensions is the detection of efforts in the 0-junctions connected to $C_{1}:m_{1}$, $C_{2}:m_{2}$ and $C_{3}:m_{3}$. Therefore, the use of the bond graph model of the distillation column allows for the detection of other variables, including chemical power.

Furthermore, the influence of some elements in the measurement of some variable in the bond graph can be obtained in a clear, simple and direct way with the use of causal paths [31].

## 7. Discussions

One of the objectives of proposing a junction structure for the class of nonlinear systems that the given distillation column represents is to verify that this graphical model effectively determines the differential equations of the system, which is demonstrated from Equations –.

The behavior of the responses of the column model has been verified according to [27,28,29]. Likewise, the following observations are given:

- Any action that causes an increase in the concentration of the most volatile component within the system automatically causes a corresponding increase in its concentration in each of the stages, which in the model corresponds to the state variables $\left(q_{1},\;q_{2},\;q_{3},\;q_{4},\;q_{5}\right)$, particularly in the variables output $q_{1}$ and $q_{5}$, which represent the mole fraction of light in the distillate and in the bottom stream.
- Any increase in feed flow, mole fraction of light component in the feed, and distillate reflux cause an increase in the state variables $\left(q_{1},\;q_{2},\;q_{3},\;q_{4},\;q_{5}\right)$ of magnitude proportional to the relative effect of each disturbance.
- Similarly, any disturbance that causes a reduction in the concentration of light, the system will decrease its concentration at each stage. The bottom current is the poorest in that component, and therefore, in the bottom reflux towards the reboiler, it should cause a decrease in the values of the state variables $\left(q_{1},\;q_{2},\;q_{3},\;q_{4},\;q_{5}\right)$.

**Figure 23:**
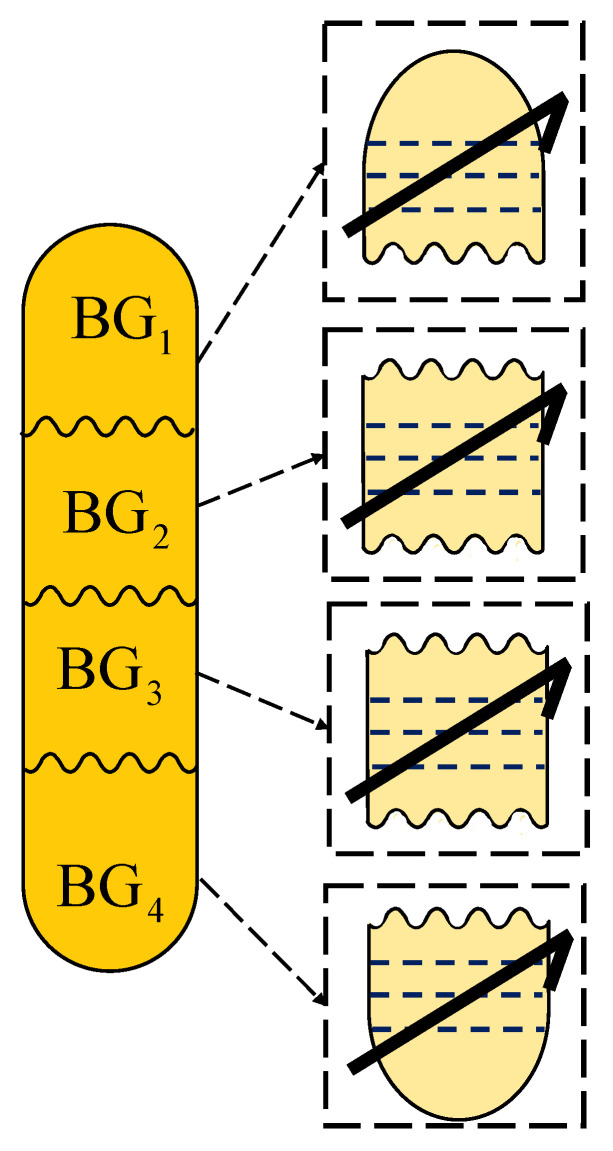
Bond graph scheme for a column of more than 5 plates.

Finally, the proposed bond graph for a five-stage column can be increased to a larger number, as illustrated in Figure 23.

According to Figure 22, there is a column of 20 plates, which is achieved by connecting four bond graphs and each of them with five plates.

## 8. Conclusions

Industrial processes generally represent nonlinear systems, whereas classical methods normally determine their behavior through nonlinear differential equations, which are complex. In this way, a graphical alternative such as the bond graph modeling of a distillation column has been presented. Due to its graphical nature, this model describes the exchange of power in the system considering energy transformation and storage.

One of the advantages of the bond graph approach in modeling the distillation columns in this paper is that it has been built in a modular way. Thus, a column formed by a large number of stages can be obtained. In order to determine the mathematical model of the column from its bond graph, a junction structure that indicates and classifies the variables and elements of the system has been proposed. Through this junction structure, a natural link of the bond graph and its mathematical representation is presented. Therefore, the structural characteristics of the system can be derived graphically and mathematically.

Other advantages of this approach are:

- If there is a reconfiguration of the column, the new bond graph is instructed to be built.
- The relationship of the variables and elements that the column contains is clear and simple with the use of causal trajectories [31], in the classical approach, it is difficult and confusing.
- The inclusion of new elements or non-modeled dynamics or the incorporation of different mathematical relationships such as a different relative volatility function is straightforward and, in the classical approach, can lead to the total reconstruction of the system.
- The analysis of the system in the bond graph is symbolic, and in the classical approach, numerical analysis is common.
- The measurement of any variable in the bond graph is straightforward, as is the chemical potential.
- The linearization of the bond graph for approximate studies can be obtained directly with [32].
- The inputs and outputs for the possible design of controllers and observers are direct in the bond graph.

Finally, the methodology exposed to two case studies has been applied. In both cases, there are binary mixtures with different relative volatilities, and the behavior of the concentration of the light component in the distillate and bottom, as well as the chemical tensions of the column, have been shown.

## Figures and Tables

**Table 1:** Power variables.

Energy Domain	Effort $\left(e\left(t\right)\right)$	Flow $\left(f\left(t\right)\right)$
Electric	Voltage	Current
Mechanics of rotation	Torque	Velocity
Mechanics of translation	Force	Velocity
Hydraulics	Pressure	Volume flow
Thermal	Temperature	Heat flow
Thermodynamics	Temperature	Entropy flow
Chemistry	Chemical tension	Molar flow

**Table 2:** Operational conditions of the distillation column.

Number of Stages, *n*	5
Feed, *F*	1 kmol/min
$m_{1}$	5 kmol
$m_{2}$	0.5 kmol
$m_{3}$	0.5 kmol
$m_{4}$	0.5 kmol
$m_{5}$	5 kmol
$z_{F}$	0.5
$L_{R}$	2.706 kmol/min
$V_{S}$	3.206 kmol/min
*B*	0.5 kmol/min
$V_{R}$	3.206 kmol/min
α	1.5

**Table 3:** Steady-state values.

$q_{1}=0.669271$
$q_{2}=0.574302$
$q_{3}=0.488711$
$q_{4}=0.412885$
$q_{5}=0.330728$

**Table 4:** Operational conditions of the distillation column.

Number of Stages, *n*	5
Feed, *F*	1 kmol/min
$m_{1}$	5 kmol
$m_{2}$	0.5 kmol
$m_{3}$	0.5 kmol
$m_{4}$	0.5 kmol
$m_{5}$	5 kmol
$z_{F}$	0.6
$L_{R}$	0.9776 kmol/min
$V_{S}$	1.5886 kmol/min
*B*	0.389 kmol/min
$V_{R}$	1.5886 mol/min
α	2.6

**Table 5:** Steady-state values.

$q_{1}=0.8472$
$q_{2}=0.6807$
$q_{3}=0.5288$
$q_{4}=0.3170$
$q_{5}=0.2118$

## Data Availability

Not applicable.

## Appendix A

**Proof.** From the seventh line of (25) with (26) and (27), the relationship between storage elements in integral and derivative causality assignment are given by

(A1) $$x_{d}\left(t\right)=F_{d}^{-1}S_{41}\left(x\right)Fx\left(t\right)$$

obtaining the derivative with respect to time,

(A2) $${\overset{•}{x}}_{d}\left(t\right)=F_{d}^{-1}{\overset{•}{S}}_{41}\left(x\right)Fx\left(t\right)+F_{d}^{-1}S_{41}\left(x\right)F\overset{•}{x}\left(t\right)$$

From the second line of (25) with (28) and (29),

$$\begin{array}{ccc} D_{in}^{l}\left(t\right) & = & {\left[I-S_{22}^{11}\left(x\right)L\right]}^{-1}\left[S_{21}^{11}\left(x\right)z\left(t\right)\right. \end{array}$$

(A3) $$\begin{array}{ccc} & & \left.+S_{22}^{12}\left(x\right)L_{m}D_{in}^{lm}\left(t\right)+S_{23}^{11}\left(x\right)u\left(t\right)\right] \end{array}$$

from the third line of (25) with (28) and (29),

$$\begin{array}{ccc} D_{in}^{lm}\left(t\right) & = & {\left[I-S_{22}^{11}\left(x\right)L_{m}\right]}^{-1}\left[S_{21}^{21}\left(x\right)z\left(t\right)\right. \end{array}$$

(A4) $$\begin{array}{ccc} & & \left.+S_{22}^{21}\left(x\right)LD_{in}^{l}\left(t\right)+S_{23}^{21}\left(x\right)u\left(t\right)\right] \end{array}$$

substituting (A4) into (A3),

(A5) $$\begin{array}{ccc} D_{in}^{l}\left(t\right) & = & {\left[I-S_{22}^{11}\left(x\right)L\right]}^{-1}\left\{S_{21}^{11}\left(x\right)z\left(t\right)+S_{23}^{11}\left(x\right)u\left(t\right)+\right. \end{array}$$

$$\begin{array}{ccc} & & \left.S_{22}^{12}\left(x\right)L_{m}{\left[I-S_{22}^{11}\left(x\right)L_{m}\right]}^{-1}\left[S_{21}^{21}\left(x\right)z\left(t\right)++S_{22}^{21}\left(x\right)LD_{in}^{l}\left(t\right)+S_{23}^{21}\left(x\right)u\left(t\right)\right]\right\} \end{array}$$

with (44), (A5) is reduced by

$$\begin{array}{ccc} D_{in}^{l}\left(t\right) & = & {\left[I-S_{22}^{11}\left(x\right)L-S_{22}^{12}\left(x\right)M_{m}\left(x\right)S_{22}^{21}\left(x\right)L\right]}^{-1}\left\{S_{21}^{11}\left(x\right)z\left(t\right)+S_{23}^{11}\left(x\right)u\left(t\right)\right. \end{array}$$

(A6) $$\begin{array}{ccc} & & \left.+S_{22}^{12}\left(x\right)M_{m}\left(x\right)\left[S_{21}^{21}\left(x\right)z\left(t\right)+S_{22}^{21}\left(x\right)M_{m}\left(x\right)S_{23}^{21}\left(x\right)u\left(t\right)\right]\right\} \end{array}$$

In a similar way, substituting (A3) into (A4),

(A7) $$\begin{array}{ccc} D_{in}^{lm}\left(t\right) & = & {\left[I-S_{22}^{22}\left(x\right)L_{m}\right]}^{-1}\left\{S_{21}^{21}\left(x\right)z\left(t\right)+S_{23}^{21}\left(x\right)u\left(t\right)+\right.\\ & & \left.S_{22}^{21}\left(x\right)L{\left[I-S_{22}^{11}\left(x\right)L\right]}^{-1}\left[S_{21}^{11}\left(x\right)z\left(t\right)+S_{22}^{12}\left(x\right)L_{m}D_{in}^{lm}\left(t\right)+S_{23}^{11}\left(x\right)u\left(t\right)\right]\right\} \end{array}$$

Equation (A7) with (43) is reduced to

$$\begin{array}{ccc} D_{in}^{lm}\left(t\right) & = & {\left[I-S_{22}^{22}\left(x\right)L_{m}-S_{22}^{21}\left(x\right)M\left(x\right)S_{22}^{12}\left(x\right)L_{m}\right]}^{-1}\left\{S_{21}^{21}\left(x\right)z\left(t\right)+S_{23}^{21}\left(x\right)u\left(t\right)+\right. \end{array}$$

(A8) $$\begin{array}{ccc} & & \left.S_{22}^{21}\left(x\right)M\left(x\right)\left[S_{21}^{11}\left(x\right)z\left(t\right)+S_{23}^{11}\left(x\right)u\left(t\right)\right]\right\} \end{array}$$

From the first line of (25) with (28), (29), (A6) and (A8),

$$\begin{array}{ccc} \overset{•}{x}\left(t\right) & = & S_{11}\left(x\right)z+S_{12}^{11}\left(x\right)L{\left[I-S_{22}^{11}\left(x\right)L-S_{22}^{12}\left(x\right)M_{m}S_{22}^{21}\left(x\right)L\right]}^{-1}\left[S_{21}^{11}\left(x\right)z\left(t\right)+\right.\\ & & \left.S_{22}^{12}\left(x\right)M_{m}\left(x\right)S_{21}^{21}\left(x\right)z\left(t\right)+S_{22}^{12}\left(x\right)M_{m}\left(x\right)S_{23}^{21}\left(x\right)u\left(t\right)+S_{23}^{11}\left(x\right)u\left(t\right)\right]\\ & & +S_{12}^{12}\left(x\right)L_{m}{\left[I-S_{22}^{22}\left(x\right)L_{m}-S_{22}^{21}\left(x\right)M\left(x\right)S_{22}^{12}\left(x\right)L_{m}\right]}^{-1}\left[S_{21}^{21}\left(x\right)z\left(t\right)\right.\\ & & \left.+S_{22}^{21}\left(x\right)M\left(x\right)S_{21}^{11}\left(x\right)z\left(t\right)+S_{22}^{21}\left(x\right)M\left(x\right)S_{23}^{11}\left(x\right)u\left(t\right)+S_{23}^{21}\left(x\right)u\left(t\right)\right] \end{array}$$

(A9) $$\begin{array}{ccc} & & +S_{12}^{13}\left(x\right)D_{out}^{nl}\left(t\right)+S_{12}^{14}\left(x\right)D_{out}^{nlm}\left(t\right)+S_{13}\left(x\right)u\left(t\right)+S_{14}\left(x\right){\overset{•}{x}}_{d}\left(t\right) \end{array}$$

with (41) and (42), (A9) is expressed by

$$\begin{array}{ccc} \overset{•}{x}\left(t\right) & = & \left\{S_{11}\left(x\right)+S_{12}^{11}\left(x\right)Q\left(x\right)\left[S_{21}^{11}\left(x\right)+S_{22}^{12}\left(x\right)M_{m}\left(x\right)S_{21}^{21}\left(x\right)\right]\right.\\ & & \left.+S_{12}^{12}\left(x\right)Q_{m}\left(x\right)\left[S_{21}^{21}\left(x\right)+S_{22}^{21}\left(x\right)M\left(x\right)S_{21}^{11}\left(x\right)\right]\right\}z+\\ & & \left\{S_{13}\left(x\right)+S_{12}^{11}\left(x\right)Q\left(x\right)\left[S_{23}^{11}\left(x\right)+S_{22}^{12}\left(x\right)M_{m}\left(x\right)S_{23}^{21}\left(x\right)\right]\right.\\ & & \left.+S_{12}^{12}\left(x\right)Q_{m}\left(x\right)\left[S_{23}^{21}\left(x\right)+S_{22}^{21}\left(x\right)M\left(x\right)S_{23}^{11}\left(x\right)\right]\right\}u\left(t\right)+ \end{array}$$

(A10) $$\begin{array}{ccc} & & +S_{12}^{13}\left(x\right)D_{out}^{nl}\left(t\right)+S_{12}^{14}\left(x\right)D_{out}^{nlm}\left(t\right)+S_{14}\left(x\right){\overset{•}{x}}_{d}\left(t\right) \end{array}$$

from (30), (31), and the fourth and fifth lines of (25), the expressions for the nonlinear dissipation fields are given by

(A11) $$\begin{array}{ccc} D_{out}^{nl}\left(t\right) & = & \phi \left(S_{21}^{31}\left(x\right)z\left(t\right)+S_{23}^{31}\left(x\right)u\left(t\right)\right) \end{array}$$

(A12) $$\begin{array}{ccc} D_{out}^{nlm}\left(t\right) & = & \phi \left(S_{21}^{41}\left(x\right)z\left(t\right)+S_{23}^{41}\left(x\right)u\left(t\right)\right) \end{array}$$

substituting (A2) into (A10) with (33), (34), (35), the state equation for this class of nonlinear systems modeled by bond graphs is proved. □
